# Modified mallampati classification in determining the success of unsedated transesophageal echocardiography procedure in patients with heart disease: simple but efficient

**DOI:** 10.1186/s12947-016-0086-z

**Published:** 2016-10-05

**Authors:** Jureerat Khongkaew, Dujdao Sahasthas, Tharrittawadha Potat, Phatchara Thammawirat

**Affiliations:** 1 Queen Sirikit Heart Center of the Northeast, Faculty of Medicine, Khon Kaen University, Khon Kaen, Thailand; 2 Division of Cardiology, Department of Medicine, Khon Kaen University, Khon Kaen, Thailand

**Keywords:** Modified Mallampati Classification, Unsedated transesophageal echocardiography, Heart disease patient

## Abstract

**Background:**

The transesophageal echocardiograhpy (TEE) has been studied worldwide. However, identifying additional factors on top of operator's experience and patient's cooperation which could influence the success of the procedure in unsedated patients with heart disease  is not well documented.

**Methods:**

Under the cross-sectional descriptive design, 85 target patients were fulfilling the criteria: being Thai national at the age of at least 20-year-old, being performed TEE by the study participant’s cardiologists, being able to communicate verbally. Seven outcomes were recorded, including gag reflex, insertion attempt, insertion time, vital signs (heart rate, oxygen saturation and mean arterial blood pressure), visible blood on TEE probe tip, and oropharyngeal pain at 1 h and 24-h.

**Results:**

There were 85 eligible patients during June 2012 to June 2013. The major participants were male (46, 54 %) and the mean age was 51.2 ± 12.5 years. The MMC class III was mostly found (33, 38.80 %). TEE probe insertion time and gag reflex were indicated statistical significance (*P* < 0.05). Linear regression revealed that MMC class III (b 3.718; SD ± 1.077; *P* = 0.001) and class IV (b 5.15; SD ± 1.286; *P* = 0.000) were statistically associated with TEE probe insertion time, whereas MMC class II was no statistically significant (b 2.348; SD ± 1.405; *P* = 0.099) according to constant value in MMC class I (5.318 s). Similarly, logistic regression indicated that the patients with high grade MMC were more likely to have gagging than the low grade MMC patients (MMC 2 OR 0.567, 95 % CI 0.09–3.42, *P =* 0.536; MMC 3 OR 5.231, 95 % CI 1.55–17.67, *P =* 0.008; MMC 4 OR 3.4, 95 % CI 0.84–13.76, *P* = 0.086).

**Conclusions:**

Modified Mallampati Classification is one of determining factors in the success of unsedated TEE procedure in patients with heart disease, especially for assessment of gagging and successful TEE probe insertion time.

## Background

In the non-invasive cardiac diagnostic settings worldwide, a transesophageal echocardiography (TEE) can be performed with or without conscious sedation. According to the guidelines for performing a TEE, the procedure is well tolerated by an unsedated patient who is adequately given oral anaesthesia [1]. Comparing with a sedated TEE, the unsedated patients show a lower incidence of cardiopulmonary complications and also receive more in benefit in terms of recovery time and medical care cost [1, 2]. However, performing a TEE without sedation requires a well cooperative patient since the procedure can easily injure organs, including lips, teeth, oropharynx, larynx, esophagus and stomach [3, 4]. In addition, the patients who show gagging during the procedure tend to have more oropharygeal injury than the absent gagging group [5, 6].

As gagging is a significant obstacle to succeed in performing an unsedated TEE, oropharynx assessment should be considered as an important process. However, previous studies mention that only operator’s experience and patient’s cooperation are the two influencing factors [1, 4]. In the field of gastrointestology, Huang, et al. compare the tolerance in esophagogastroduodenoscopy (EGD) among the patients based on Modified Mallampati Classification (MMC) [6]. The result clearly shows that the patients with MMC class III and class IV mostly present gagging during the procedure which leads the patient to be intolerant and be given sedation. Also, in the field of anaesthesiology, the MMC has been accepted as one of the factors affecting a successful endotracheal tube intubation [7, 8, 9]. Focusing on the field of cardiology, there is a lack of data supporting the correlation between MMC and the TEE outcomes. Even though TEE probe insertion is technically easier than endotracheal tube intubation, some complications can occur since the long probe has to be passed oropharynx before being inserted into the esophagus. From this point of view, our present study aims to identify additional factors on top of operator experience and patient co-operation which can influence the success of a TEE procedure in unsedated patients with heart disease.

## Methods

### Population

This study was approved by the Human Research Ethics Committee of Khon Kaen University, Thailand. The patients who were considered for the study’s inclusion would meet specific criteria, including being a Thai national at the age of 20-year-old or more, being performed the TEE by the participant’s cardiologist, being able to communicate verbally in Thai language, and willing to have the unsedated TEE as well as willing to be the study’s participant. The excluded patients were those younger than 20-year-old, unwilling to have the unsedated TEE and to participate in the study, incomplete informed consent form, unable to communicate verbally in Thai language, having a history of dysphagia or bleeding disorder, undergoing oropharyngeal surgery, unable to be assessed MMC, and being given sedation before or during the procedure.

### Data collection

**Fig. 1:**
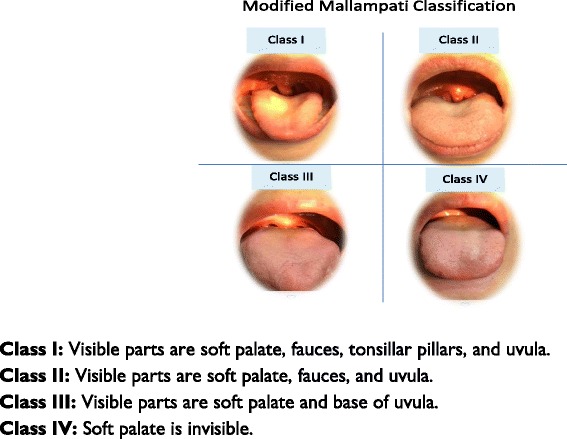
Modified Mallampati Classification

The data was collected using the specific form, consisted of three significant parts: demographic data, factors involving TEE procedure, and the seven TEE outcomes (insertion attempt, successful insertion time, gag reflex during insertion, vital signs’ change, oropharyngeal pain at 1-h and 24-h, and visible blood on probe tip). Initially, the informed consent form must be completed. Throughout the procedure, neither the cardiologists together with the two collecting data nurses nor the patient themselves knew the patient’s MMC class, except the two well-trained MMC assessment nurse who graded patients’ MMC class using the MMC chart as shown in Fig. 1.

According to our hospital TEE preparation, the patient would be orally anaesthetized receiving lidocaine (Astra Zeneca) both 10 % spray and 2 % jelly. With a total safe dosage of less than 400 mg [10], 150 mg of lidocaine jelly was orally given to the patient twice; the second dose was administered five minutes following the first. The patient would be then evaluated the gag reflex and would be given 2 more puffs (20 mg) of lidocaine spray if gagging was presented. When the oropharyngeal preparation was completed, the patient was placed in left lateral decubitus position.

Before TEE probe insertion, a bite guard was already put in place. While the patient was lying in the specific position under the safety setting, the operator gently entered a lubricated TEE probe (model GE 6Tc) into the patient oral cavity. Once the probe being passed through the patient’ mouth until being placed into the esophagus, presented gagging, vital signs, successful TEE probe insertion time and attempt were noted in agreement of the two collecting data nurses.

**Fig. 2:**
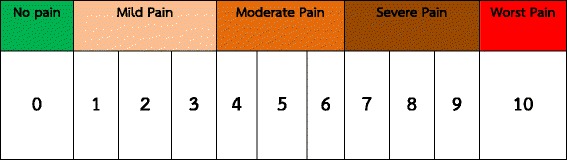
Oropharyngeal pain scale, adapted from visual analog scale (VAS)

After the procedure had been completed, the transducer was slowly pulled out of the patient mouth and was placed on a white towel in order to evaluate blood on the transducer tip. The patient vital signs were continuously monitored for 30 min. Oropharyngeal pain at 1 h and 24-h were assessed by means of a phone call asking the patient to state a 0–10 oropharyngeal pain score, adapted from visual analog scale (VAS) as shown in Fig. 2.

### Definition of terms

1. Patient’s cooperation refers to a willingness to have unsedated TEE procedure which is evaluated by observation of the patient’s compliance with topical anaesthesic agent given and facial expression. The criteria are below.
  1.1 Excellent cooperation is rated for the patient who shows smiling face and truly willingness to be anaesthetized for the unsedated TEE procedure.
  1.2 Good cooperation is described as the patient presents with neutral face and actions.
  1.3 Poor cooperation refers to the patient showing unhappy face and being difficult to give topical anaesthesic agent.
2. Insertion attempt means the number of attempt to insert the TEE probe into the patient’s esophagus successfully.
3. Successful probe insertion time refers to the specific time when the TEE probe can be in place.
4. Gag reflex during insertion refers to a gagging which is stimulated by the touching of the TEE probe on the patient’s oropharynx before being placed into the esophagus.
5. Vital signs’ change is noted if there is a decrease of oxygen saturation less than 90 % or a 20 % change of either heart rate (HR) or mean arterial pressure (MAP).

### Statistical analysis

The raw data was analyzed using SPSS for windows version 17.0. The continuous data were presented as mean ± standard deviation (SD) while all categorical data were shown as absolute number and percentage (%). The difference and correlation between the MMC and seven related variables (insertion attempt, successful probe insertion time, gag reflex, vital signs’ change, visible blood on probe tip, and oropharyngeal pain score at 1-h and at 24- h) were analyzed using one-way ANOVA and simple linear regression analysis for continuous data, whereas chi square and logistic regression analysis were used for analyzing categorical variables. *P* value < 0.05 was considered as statistic significant.

## Results

### Patient’s demographic characteristics

Throughout a year (June 2012-June 2013), a total of 147 heart disease patients underwent the TEE procedure at Queen Sirikit Heart Center of the Northeast, Faculty of Medicine, Khon Kaen University. There were 86 patients who met the study inclusion criteria. Only one case was excluded due to left jaw pain which affected mouth opening. Out of 85 eligible patients, most of them were male (46, 54 %). The mean age and BMI were 51.2 ± 12.48 and 23.95 ± 4.72, respectively. Sixty-one patients (71.8 %) had no experience with the TEE, but showed good cooperation (59, 64.40 %). Also, nearly half of them took anticoagulant medications (42, 49.40 %). MMC class III was the most presented class (33, 38.80 %) and was mostly found in women (20, 60.6 %). All parameters are shown in Table 1.

**Table 1:** Patient’s demographic characteristics

Demographic Characteristics	Total (*n* = 85)	MMC 1 (*n* = 22)	MMC 2 (*n* = 14)	MMC 3 (*n* = 33)	MMC 4 (*n* = 16)
MMC class No. (%)	85 (100)	22 (25.9)	14 (16.5)	33 (38.8)	16 (18.8)
Age (Mean ± SD)	51.20 ± 12.48	48.82 ± 14.53	50.86 ± 10.54	49.12 ± 12.05	59.06 ± 9.33
BMI (Mean ± SD)	23.95 ± 4.72	23.20 ± 3.50	23.20 ± 3.60	24.35 ± 5.40	24.90 ± 5.70
Gender- No. (%)					
Male	46 (54.1)	14 (63.6)	9 (64.3)	13 (39.4)	10 (62.5)
Female	39 (45.9)	8 (36.4)	5 (35.7)	20 (60.6)	6 (37.5)
Education - No. (%)					
Elementary	47 (55.3)	11 (50.0)	8 (57.1)	17 (51.5)	11 (68.8)
High School	18 (21.2)	8 (36.4)	2 (14.3)	7 (21.2)	1 (6.3)
Higher Education	20 (23.5)	3 (13.6)	4 (28.6)	9 (27.3)	4 (25.0)
Previous TEE - No. (%)					
Yes	24 (28.2)	5 (22.7)	4 (28.6)	9 (27.3)	6 (37.5)
No	61 (71.8)	17 (77.3)	10 (71.4)	24 (72.7)	10 (62.5)
Cooperation - No. (%)					
Poor	5 (5.9)	2 (9.1)	1 (7.1)	2 (6.1)	0 (0.0)
Good	59 (69.4)	14 (63.6)	11 (78.6)	26 (78.8)	8 (50.0)
Excellent	21 (24.7)	6 (27.3)	2 (14.3)	5 (15.2)	8 (50.0)
Anticoagulation - No. (%)					
Yes	42 (49.4)	14 (63.6)	4 (28.6)	12 (36.4)	12 (75.0)
No	43 (50.6)	8 (36.4)	10 (71.4)	21 (63.6)	4 (25.0)

### Comparison of TEE outcomes among the patients based on MMC

Out of the seven outcomes, only the gag reflex and the successful TEE probe insertion time indicated statistical significance (*P* = 0.005). Among the four groups, the patient with MMC class III (20, 60.6 %) and MMC IV (8, 50.0 %) were the first two group which mostly presented gagging while the less presented gagging were the patients with MMC class I (5, 22.7 %) and class II (2, 14.3 %). Similar to gag reflex, the patients with MMC class III and class IV (9.04 ± 3.72, 10.48 ± 6.53) had longer successful insertion time than the patients with MMC class I and class II (5.32 ± 1.67, 7.67 ± 2.50). Contrary to the insertion attempt, although MMC class IV showed the highest number of attempt (1.38 ± 1.09), the differences number of attempt among MMC classes showed no statistical significance (*P* = 0.133).

Focusing on the vital signs’ change, there was no statistically significant difference between MMC and each of the three vital signs, including mean arterial pressure (MAP), heart rate (HR), and oxygen saturation (O2sat). However, the percentage of HR change was increased in each higher MMC classes as follows: MMC class I was 36.36 %, MMC class II was 42.90 %, MMC class III was 45.45 % and MMC class IV was 56.25 %. Also, the MAP in the patients with MMC class III (6, 18.18 %) and MMC class IV (5, 31.25 %) showed higher percentages than the patients with MMC class I (2, 9.10) and MMC class II (1, 7.14 %) while there was an unremarkable change of the O2sat (≤90 %) throughout the procedure.

The last three outcomes, recorded after pulling the TEE probe out of the patient’s mouth were oropharyngeal pain (OP) at 1-h, oropharyngeal pain (OP) at 24-h, and visible blood on probe tip. According to the OP score 0–10, the mean score of both OP at 1 h (1.31 ± 1.23) and 24-h (0.78 ± 1.15) showed mild pain score and no statistically significant difference (*P* = 0.086, *P* = 0.950). Likewise, 21 (24.71 %) patients were found to have blood on probe tip as well as no statistically significant difference (*P* = 0.983). The data are presented in Table 2.

**Table 2:** Comparison of TEE outcomes among Modified Mallampati Classification

Outcomes	Total (*n* = 85)	MMC 1 (*n* = 22)	MMC 2 (*n* = 14)	MMC 3 (*n* = 33)	MMC 4 (*n* = 16)	P-value
Gag Reflex - No. (%)	35 (41.18)	5 (22.7)	2 (14.3)	20 (60.6)	8 (50.0)	0.005
Attempt (Mean ± SD)	1.12 ± 0.52	1.14 ± 00.35	1.00 ± 0.00	1.03 ± 0.03	1.38 ± 1.09	0.133
Time (Mean ± SD)	8.13 ± 4.28	5.32 ± 1.67	7.67 ± 2.50	9.04 ± 3.72	10.48 ± 6.53	0.003
Vital Signs’ Change						
MAP - No. (%)	14 (16.47)	2 (9.10)	1 (7.14)	6 (18.18)	5 (31.25)	0.224
O2sat - No. (%)	0 (0.0)	0 (0.0)	0 (0.0)	0 (0.0)	0 (0.0)	-
HR - No. (%)	38 (44.71)	8 (36.36)	6 (42.90)	15 (45.45)	9 (56.25)	0.680
Throat Pain Score						
1-h (Mean ± SD)	1.31 ± 1.23	1.09 ± 0.97	1.29 ± 1.54	1.55 ± 1.28	1.13 ± 1.20	0.086
24-h (Mean ± SD)	0.78 ± 1.15	0.64 ± 1.23	0.57 ± 1.02	1.06 ± 1.17	0.56 ± 1.09	0.950
Bleeding - No. (%)	21 (24.71)	5 (22.73)	4 (28.57)	8 (24.24)	4 (25.00)	0.983
Operator A	38 (44.70)	11 (28.95)	5 (13.16)	15 (39.48)	7 (18.43)	
Insertion attempt	1.05 ± 0.23	1.09 ± 0.30	1.00 ± 0.00	1.07 ± 0.26	1.07 ± 0.26	0.810
Insertion time	6.85 ± 2.37	4.82 ± 1.25	7.80 ± 2.59	7.95 ± 2.03	7.00 ± 2.58	0.003
Gag Reflex	16 (42.11)	3 (27.28)	0 (0.0)	10 (66.67)	3 (42.86)	0.039
Bleeding	5 (13.16)	1 (9.09)	1 (20.00)	1 (6.67)	2 (28.58)	0.499
Operator B	47 (55.30)	11 (23.40)	7 (14.90)	20 (42.56)	9 (19.14)	
Insertion Attempt	1.15 ± 0.68	1.18 ± 0.41	1.00 ± 0.00	1.00 ± 0.00	1.67 ± 1.41	0.085
Insertion Time	9.21 ± 5.18	5.82 ± 1.94	7.57 ± 2.64	9.94 ± 4.56	13.18 ± 7.50	00.007
Gag Reflex	22 (48.90)	2 (18.19)	1 (12.50)	13 (72.23)	6 (66.67)	0.004
Bleeding	16 (34.79)	4 (36.37)	3 (37.50)	7 (38.89)	2 (22.23)	0.850

### Correlation between the outcomes and the MMC

Having been identified as statistically significant variables, gag reflex and successful TEE probe insertion time were further analyzed using regression analysis.

Based on logistic regression, comparing gag reflex between MMC class I and the others, while the patients with MMC class II were indicated non statistical significance (OR 0.567; 95 % CI 0.094–3.423; *P* = 0. 536), the high grade MMC such as class III was found to be statistically significant associated with gag reflex during the TEE probe insertion (OR 5.231; 95 % CI 1.548–17.670; *P* = 0.008). Moreover, although the association was no statistically significant, the patients with MMC class IV also had a tendency to have gagging (OR 3.4; 95 % CI 0.840–13.761; *P* = 0.086). The data is shown in Table 3.

**Table 3:** Correlation between MMC and gag reflex

					95 % CI	
MMC	Wald	df	P-value	Exp (B)	Lower	Upper
MMC 1	11.640	3	0.009	1		
MMC 2	0.383	1	0.536	0.567	0.094	3.423
MMC 3	7.097	1	0.008	5.231	1.548	17.670
MMC 4	2.934	1	0.086	3.400	0.840	13.761
Constant	5.786	1	0.016	0.294		

Simple linear regression was performed in order to identify the association between successful TEE probe insertion time and the MMC as shown in Table 4. By using the successful time of MMC class I as the constant (5.318 s), the results indicated that the high grade MMC class III (b 3.718; SD ± 1.077; *P* = 0.001) and IV (b 5.15; SD ± 1.286; *P* = 0.000) were statistically significant correlated with the successful TEE probe insertion time, whereas the patients with MMC class II were no statistically significant (b 2.348; SD ± 1.405; *P* = 0.099). The data is shown in Table 4.

**Table 4:** Association between successful TEE probe insertion time and MMC

MMC	B	SD	Mean + SD	Mean-SD	P-value
Constant	5.318	0.835	6.02	4.35	0.000
MMC 2	2.348	1.405	3.76	0.90	00.099
MMC 3	3.718	1.077	4.80	2.65	0.001
MMC 4	5.517	1.286	6.45	3.88	0.000

## Discussion

Based on the study results, the high grade MMC (class III and class IV) was statistically significant associated with the gag reflex and the insertion time. These findings are additional clinical information for performing a TEE since previous studies mention only operator’s experience and patient’s cooperation as the key success factors [1, 4]. However, one of the most important problems in performing a TEE is insertion of the probe, especially in the unsedated patients.

During the TEE probe insertion, even though topical anaesthetic agent has been applied throughout the oropharynx, gagging still remains in some cases. This physical reaction is induced by the touch of the transducer on any six sensitive oropharyngeal parts, including soft palate, uvula, fauces, posterior pharyngeal wall, back of the tongue and epiglottis [5, 6]. The effect of gagging can cause a failure of the probe insertion or aspiration during the procedure [5]. As reported by Huang, et al., the patients who have gagging are tended to have lower tolerance for esophagogastroduodenoscopy (EGD) than the patients in the opposite group. They also find out that the patients with high grade MMC (classes III and IV) are found to have more gagging than the low grade MMC patients (classes I and II) [6]. In agreement with our results, the patients who presented with MMC class III and class IV had a 5.2–fold and 3.4-fold more gagging than MMC class I patients. This finding was similar to the insertion time which also associated with MMC.

In reference to our results, the mean time of the fastest probe insertion was 5.32 ± 1.67 s which was found in the group of MMC I while the other three groups of the higher classes showed longer times as in MMC class II was 2.35 ± 2.5 s (*P* = 0.099), MMC class III was 3.72 ± 3.72 s (*P* = 0.001) and MMC class IV was 5.16 ± 6.53 s (*P* = 0.000).

Therefore, according to regression equation, Y = ax + b [23, 24], the successfully inserted time of the patients with MMC class II, class III and class IV are as follows: 7.67 ± 2.5 s, 9.04 ± 3.72 s, and 10.48 ± 6.53 s. Comparing to the another study, there is a lack of data on the TEE probe insertion time, but an approximation is within 1 min [25].

To the best of our knowledge, even though all participants were successfully performed the TEE without sedation, MMC should be considered as one of determining factors affecting the unsedated TEE’s outcome since it is related to gagging and probe insertion time. These correlations may be explained using MMC criteria classified by oropharyngeal cavity [11–16]. By the view of fully opened mouth and protruded tongue without any sounds, MMC class III and class IV allow the examiner to see only soft palate and maybe uvular because the size and position of the tongue which are larger and farther than MMC class I and class II [12]. This specific anatomy is an obstacle to performing the TEE because of the compression of the probe which spontaneously creates a direct pressure on the posterior of the tongue leading to a spasm of the pharynx, a natural mechanism of choking prevention [17–20]. Moreover, the narrow oropharyngeal cavity also affects the procedure in terms of difficulty passing the TEE probe into esophagus. For these two reasons, the patients with the narrow oral cavity (MMC class III and class IV) are tended to experience longer successful insertion time than those who have wider oral cavity (MMC class I and class II).

The other interesting finding was the patients with MMC class III and class IV had a tendency to have oropharyngeal pain at 1 h after the procedure (*P* = 0.086). This result could be explained based on the successful insertion time and gagging which were related to MMC. As mentioned above, the patients with high grade MMC had narrow oral cavity which might be abraded easily on oropharyngeal mucous membrane by the TEE probe during insertion, especially when having gagging. That is, the patients who present more gagging during the TEE procedure are likely to experience more oropharyngeal pain at 1 h after the procedure than others [25–27]. This finding supports the TEE is not only a safe procedure but also a non-admitted procedure. According to the TEE guideline, an outpatient can be discharged if there is non-serious complication after the procedure [28].

The reduction of gagging during performing endoscopic procedure has been studied worldwide in order to increase patients’ tolerance and comfort [14, 15],such as using a micro TEE probe and intra cardiac echocardiography probe (ICE) instead of using a conventional probe [21, 22]. Moreover, Tsuboi et al., claim that performing an unsedated EGD by passing the EGD probe through nasal cavity shows better outcomes than passing through oral cavity [12]. Apart from the equipment and the passage, Ulusoy and Kucukarslan state that the sitting position can help the patient to be successfully inserted the TEE probe [6]. Similar to Samsoon and Young, in the field of anesthesiology, neck flexion and head extension are the two important factors facilitating the operator to successfully intubate endotracheal tube.

However, in a busy non-invasive cardiac testing setting or a non-anesthesiology setting, the TEE may be performed without sedation as well as using the conventional probe and technique. In such a limited resource setting, MMC can be used for a quick assessment of gagging which will be helpful in terms of administrating topical anaesthesia. Moreover, the patients with MMC class III and class IV may need to be placed in a particular position of head and neck instead of placing them on the conventional left lateral decubitus position which focuses only on aspiration prevention [22]. In summary, optimizing the unsedated TEE outcomes, the patients with high grade MMC should be given effective oropharyngeal anaesthesia and be placed in a proper position.

### Limitations of the study

The three main points being considered as the study limitations are sample size, other factors affecting gagging, and the subjects’ age. First, our data were unavoidably analyzed from a small number of patients from single heart center and the totally unequal subject numbers in each group. Further study may need to investigate in a larger sample size. Next, the other factors affecting gaging apart from the MMC were not included in the study protocol. These factors may also affect gagging during TEE probe insertion in the patients with MMC classes I and II. Last, our results might not be generally used as a reference for the heart disease patients of all ages because most participants were middle aged and cooperative.

## Conclusion

Our study demonstrates that MMC is positively associated with the successful TEE probe insertion time. Moreover, the high grade MMC patients (MMC class III and class IV) are found to be correlated with gagging during the TEE probe insertion and found to have a tendency toward oropharyngeal pain at 1 h after the TEE. From these reasons, MMC should be considered as one of determining factors in the success of unsedated TEE procedure in the patients with heart disease. Therefore, in order to optimize unsedated TEE outcomes, the patients should be assessed MMC which will benefit in terms of administrating topical anaesthesia.

## Abbreviations

EGD: Esophagogastroduodenoscopy
HR: Heart rate
ICE: Intra cardiac echocardiography probe
MAP: Mean arterial pressure
MMC: Modified mallampati classification
SD: Standard deviation
TEE: Transesophageal echocardiography
TN-EGD: Transnasal esophagogastroduodenoscopy
TO-EGD: Transoral esophagogastroduodenoscopy
