# Erratum to: Modified mallampati classification in determining the success of unsedated transesophageal echocardiography procedure in patients with heart disease: simple but efficient

**DOI:** 10.1186/s12947-017-0095-6

**Published:** 2017-03-13

**Authors:** Jureerat Khongkaew, Dujdao Sahasthas, Tharrittawadha Potat, Phatchara Thammawirat

**Affiliations:** 1 Queen Sirikit Heart Center of the Northeast, Faculty of Medicine, Khon Kaen University, Khon Kaen, Thailand; 2 Department of Medicine, Division of Cardiology, Khon Kaen University, Khon Kaen, Thailand

In this version of this article that was originally published [1] there is an error in the Results section whereby “during June 2012 to June 2013” should have been “during June 2013 to June 2014”. Therefore the sentence “There were 85 eligible patients during June 2012 to June 2013.” Should have been “There were 85 eligible patients during June 2013 to June 2014.” There is also the same error in the text and so “Throughout a year (June 2012-June 2013), a total of 147 heart disease patients underwent the TEE procedure at Queen Sirikit Heart Center of the Northeast, Faculty of Medicine, Khon Kaen University.” Should be “Throughout a year (June 2013-June 2014), a total of 147 heart disease patients underwent the TEE procedure at Queen Sirikit Heart Center of the Northeast, Faculty of Medicine, Khon Kaen University.” The original article has now been corrected.
